# Contemporary clinical applications of venous excess ultrasound: A scoping review protocol

**DOI:** 10.1371/journal.pone.0340162

**Published:** 2025-12-31

**Authors:** Shoheb Hassan, Ali Hassan

**Affiliations:** 1 Aston Medical School, College of Health and Life Sciences, Aston University, Birmingham, United Kingdom; 2 University of Exeter Medical School, Faculty of Health and Life Sciences, University of Exeter, Exeter, United Kingdom; Ataturk University Faculty of Medicine, TÜRKIYE

## Abstract

**Objective:**

This scoping review aims to synthesise the primary literature on the current and emerging clinical applications of venous excess ultrasound, with a particular interest in its use beyond acute and critical care contexts.

**Introduction:**

Venous excess ultrasound is a novel point-of-care ultrasound tool designed to assess systemic venous congestion. While it has been widely adopted in critical and acute care settings, increasing clinical interest suggests broader diagnostic, prognostic, and therapeutic utility beyond these contexts. Despite this, no comprehensive synthesis has yet mapped the full range of its clinical applications.

**Inclusion Criteria:**

We will include primary studies involving patients of any age or clinical indication in which venous excess ultrasound has been applied in clinical care. Only studies published in English from 1 January 2020 onward will be considered. Inclusion is limited to studies reporting on the clinical utility of venous excess ultrasound. Non-human studies, abstract-only publications, secondary reviews, and purely technical imaging studies without clinical application will be excluded.

**Methods:**

Following PRISMA-ScR guidelines, this scoping review will conduct systematic searches of both peer-reviewed and grey literature sources, including PubMed, Scopus, the Cochrane Library, ProQuest, Google Scholar, preprint servers, and clinical trial registries. Where literature sources allow, searches will be limited to studies written in English and published from 1 January 2020 onward. References will be managed and deduplicated using Zotero. Full-text screening and data extraction will be performed independently by two reviewers using Rayyan and standardised Microsoft Excel forms. Disagreements will be resolved through discussion or, if necessary, by a third reviewer. Results will be summarised in tables and narratively.

## Introduction

Systemic venous congestion is a functional result of elevated central venous pressure that impairs visceral organ perfusion. It has emerged as a critical contributor to organ dysfunction across a range of clinical conditions, including acute kidney injury (AKI), heart failure, fluid overload, and multi-organ dysfunction syndromes [1]. Venous congestion is increasingly recognised in both acute and chronic care settings not merely as a passive consequence of disease, but as a dynamic, potentially modifiable pathophysiological process that directly compromises organ perfusion and function. Despite growing awareness, traditional assessments such as physical examination are limited by patient body habitus, clinician expertise, and low sensitivity. Inferior vena cava ultrasound has also shown promise in quantifying venous congestion; however, studies suggest limited clinical utility when used in isolation [2]. These limitations underscore the need for more reliable and accessible bedside tools to evaluate venous congestion in clinical settings.

In 2020, Beaubien-Souligny et al. introduced venous excess ultrasound (VExUS), a Doppler-based ultrasound grading system designed to quantify systemic venous congestion through non-invasive assessment of the inferior vena cava, hepatic, portal, and intrarenal veins [3]. This composite score categorises the severity of venous congestion to support clinical decision-making, particularly in critically ill patients.

Since its inception, VExUS has been studied in a variety of clinical contexts, including critical care, emergency medicine, and acute medicine [4–6]. For example, it has shown promise in predicting adverse outcomes such as AKI and has been used to monitor AKI resolution [7,8]. In addition, VExUS may guide and assess responses to fluid status interventions [9].

A prior systematic review by Gupta et al. synthesised evidence on the clinical utility of VExUS, focusing specifically on perioperative care, intensive care, and emergency department settings [10]. More recently, a scoping review protocol registered on OSF—titled *“The Utility of VExUS in the Critically Ill”*—centres on evaluating VExUS as a tool for fluid status assessment and management in ICU populations [11]. Although informative, both reviews adopt specifically focused scopes in terms of population and clinical contexts, leaving broader applications of VExUS underexplored. To ensure no recent reviews overlap with our scope, we conducted a preliminary search of PubMed, the Cochrane Database of Systematic Reviews, PROSPERO, Open Science Framework, and JBI Evidence Synthesis. There were no current or in-progress systematic or scoping reviews that aim to *comprehensively* map the clinical application of VExUS, particularly regarding its use beyond acute and critical care settings. This highlights a significant evidence gap, especially given the growing interest in extending VExUS to broader clinical contexts.

Given this context, a scoping review is the most appropriate methodological approach for the current state of the literature. Scoping reviews are particularly well-suited to exploring the breadth and depth of emerging evidence, identifying knowledge gaps, and informing future research priorities—especially in fields where studies are heterogeneous in design, purpose, and outcome measures. This approach aligns with the complex and evolving nature of the VExUS literature, where variability in implementation is as relevant as outcome data itself.

This scoping review aims to map the primary literature on VExUS use across diverse clinical contexts and patient populations, with particular interest in identifying whether and how VExUS has been applied outside the acute and critical care environments. Our review adopts a broader scope intended to complement, rather than duplicate, ongoing work. Furthermore, our search strategy includes grey literature and captures studies published up to the date of the upcoming search, thereby offering an up-to-date and more inclusive synthesis. Notably, the Gupta et al. review concluded its search in May 2023 and did not report inclusion of grey literature sources [10].

## Research questions

The research questions for the scoping review are as follows. Question 2 is considered a focused sub-analysis within the broader scope of Question 1.

1. In which clinical contexts has the utility of VExUS been studied?
2. How has the primary literature explored the use of VExUS outside of the critical care or acute medical context?
3. What emerging or proposed clinical applications of VExUS are being explored in ongoing trials or future research directions?

## Inclusion criteria

### Participants

This review will include studies involving patients of any age or demographic in whom VExUS has been explicitly applied and reported. Eligible participants may be drawn from any clinical setting, including but not limited to intensive care units (ICU), perioperative care, emergency departments, general hospital wards, or outpatient environments. There will be no exclusions based on the underlying condition or diagnosis, provided that the VExUS system was used in a clinical context.

### Concept

The concept of interest is the application of the VExUS scoring system to assess systemic venous congestion or related hemodynamic parameters. Studies will be included if they describe clinical uses of VExUS, including diagnostic, therapeutic, or prognostic applications—such as guiding fluid management, risk stratification, or informing treatment decisions.

### Context

The review will consider studies from all clinical environments where VExUS has been used, without restriction by healthcare setting, geographic region, or patient population. This includes acute, subacute, and outpatient settings.

### Types of sources

Eligible study designs will include primary clinical research such as observational studies (prospective or retrospective), interventional trials, case series, and case reports. Grey literature sources reporting primary data—such as registered clinical trial protocols, dissertations, and other unpublished materials—will also be included. Studies will be included regardless of the degree of methodological or outcome reporting, though such limitations will be noted during data synthesis. Only studies published in English will be included. The date range for inclusion is from January 1, 2020, to the date of the search.

### Exclusion criteria

- Non-human or animal studies.
- Records available only as abstracts, including conference proceedings and posters without full texts.
- Editorials, letters, narrative reviews, systematic reviews, and meta-analyses without original primary data.
- Studies focused exclusively on non-clinical or purely technical imaging methods not related to clinical application of VExUS.

## Methods

To ensure methodological rigor and reproducibility, this review was developed in accordance with the PRISMA Extension for Scoping Reviews (PRISMA-ScR) [12], and PRISMA Protocol (PRISMA-P) 2015 checklist [13], further guided by the Joanna Briggs Institute Manual for Evidence Synthesis [14]. Completed PRISMA-ScR (S1 File) and PRISMA-P (S2 File) checklists are available as supporting information.

The review is prospectively registered on the Open Science Framework (OSF), consistent with best practices for protocol transparency. Further procedural details, including the full data extraction form and protocol updates, are available on our OSF registration (osf.io/64abk). We will employ an adaptive approach, allowing methodological modifications in response to procedural challenges, with all changes transparently documented through timely updates on the OSF.

### Search strategy

Following PRISMA-ScR guidelines, we will conduct systematic searches for both peer-reviewed and grey literature on the clinical applications of the Venous Excess Ultrasound Score (VExUS), focusing on studies published from 1 January 2020 onwards. Our search will encompass a range of databases, including PubMed, Scopus, Cochrane Library, and ProQuest. To ensure comprehensive coverage of grey literature, we will also search additional sources such as Google Scholar, preprint servers (medRxiv, bioRxiv), and clinical trial registries including ClinicalTrials.gov and the World Health Organization (WHO) International Clinical Trials Registry Platform.

An initial limited search of PubMed was undertaken to identify relevant terminology and indexing patterns. Keywords identified in titles and abstracts were then used to develop tailored search strategies for each database. We tailored searches to each database’s indexing structure, as outlined in Table 1.

**Table 1:** Search strategy used across databases.

Database	Refined Search Strategy	Search Limits (where platforms support filtering)
PubMed	“venous excess ultrasound score” OR “VExUS score” OR “venous excess ultrasound” OR VExUS	Date: 1 Jan 2020 – date of search; Language: English
Scopus	“venous excess ultrasound score” OR “VExUS score” OR “venous excess ultrasound” OR VExUS	Date: 1 Jan 2020 – date of search; Language: English
Cochrane Library	“venous excess ultrasound score” OR “VExUS score” OR “venous excess ultrasound” OR VExUS	Date: 1 Jan 2020 – date of search; Language: English
Google Scholar	“venous excess ultrasound score” OR “VExUS score” OR “venous excess ultrasound” OR VExUS	Date: 1 Jan 2020 – date of search; Language: English
ProQuest	“venous excess ultrasound score” OR “VExUS score” OR “venous excess ultrasound” OR VExUS	Date: 1 Jan 2020 – date of search; Language: English
bioRxiv & medRxiv	Separate searches with each term: (1) “venous excess ultrasound score”, (2) “VExUS score” (3) “venous excess ultrasound”, (4) VExUS	Date: 1 Jan 2020 – date of search; Language: English
ClinicalTrials.gov	“venous excess ultrasound score” OR “VExUS score” OR “venous excess ultrasound” OR VExUS	Date: 1 Jan 2020 – date of search; Language: English
WHO International Clinical Trials Registry Platform	“venous excess ultrasound score” OR “VExUS score” OR “venous excess ultrasound” OR VExUS	Date: 1 Jan 2020 – date of search; Language: English

To balance search precision with comprehensiveness, we applied database-specific search strategies (e.g., in PubMed, Scopus, and Cochrane Library) that included studies in which the terms “venous excess ultrasound score,” “VExUS score,” “venous excess ultrasound,” or “VExUS” appeared in any searchable field (e.g., title, abstract, or full text). Given the specificity of the concept, broader terms such as “ultrasound” or “venous congestion” were intentionally excluded to minimise the retrieval of irrelevant records. As no relevant MeSH terms currently exist for these concepts, keyword-based strategies were used across all databases. This approach ensures the inclusion of studies focused on the clinical application of VExUS. By searching for these keywords across all fields, we aim to capture studies in which VExUS is discussed only in the methods or full text. To further enhance coverage, we supplemented our search with grey literature. We consider this strategy appropriate for identifying studies where VExUS plays a central role in clinical evaluation and outcomes, consistent with our eligibility criteria.

Given the specificity of the VExUS terminology and our inclusion of full-text and grey literature searches across multiple platforms, we anticipate a low yield from citation tracking. However, if clusters of key studies emerge during synthesis, we may revisit citation tracking selectively at that stage. This flexibility will ensure that we are responding appropriately to the literature that is available to us.

Two reviewers will independently pilot the search strategies to assess comprehensiveness and reproducibility prior to full execution.

Due to institutional access differences across ProQuest’s federated databases, search results may vary slightly depending on database availability. To minimise variability, all ProQuest searches were conducted from University of Exeter with the same database selection and search filters.

### Study/source of evidence selection

All identified citations from the search will be collated and imported to Zotero (version 7.0.15) for reference management. Duplicates will be removed using both automated and manual processes.

We will conduct screening at the full-text level only, for two key reasons. First, a preliminary trial of our search strategy suggests that the number of records remaining after de-duplication will be relatively small, making full-text screening logistically feasible. More importantly, we recognise that some studies may describe the clinical use of VExUS without explicitly mentioning it in the title or abstract. Relying on title and abstract screening alone could therefore lead to the inadvertent exclusion of relevant studies. Full-text screening maximises sensitivity and ensures that studies reporting on VExUS, even if not indexed under that term, are appropriately considered for inclusion.

Following de-duplication, a pilot screening of a random sample of 20 full texts will be conducted independently by two reviewers to assess inter-reviewer agreement against the eligibility criteria. Disagreements will be discussed, and only necessary modifications will be made to the eligibility criteria, to improve consistency. Formal screening will commence once an agreement threshold of ≥80% is achieved. All citations will be retrieved and imported into Rayyan, where independent screening of full-text articles will be conducted by two reviewers. Reasons for exclusion at full-text screening will be recorded.

Reviewers will resolve discrepancies at any screening stage through discussion. If consensus cannot be reached, a third reviewer will adjudicate. Inter-rater reliability (e.g., Cohen’s kappa) will be calculated to quantify the level of agreement between reviewers.

Final screening outcomes and data will be stored securely in Microsoft Excel spreadsheets, with routine backups maintained. The search and selection process will be reported in full and presented using a PRISMA flow diagram [12].

### Data extraction

Data will be extracted from included full-text papers, by two independent reviewers, using structured Microsoft Excel forms. The data extracted will include details outlined in our data extraction chart (Table 2). Where studies report incomplete or missing data, we will note these explicitly during extraction and synthesis. Prior to commencing formal data extraction, two reviewers will pilot the data extraction process on 10 randomly selected articles, using the data extraction chart. Both reviewers will discuss disagreements in effort to establish a consistent pattern of extraction. The data extraction chart will be modified and revised as necessary during this process. Modifications will be documented in the final review.

**Table 2:** Preliminary data extraction chart.

Variable	Description/ Notes
Author(s)	Main author(s)
Source Type	Journal article, preprint, thesis, grey or peer-reviewed literature
Year	Year of publication or availability
Country	Country where study was conducted
Study Design	e.g., RCT, observational, case series, case report, interventional
Sample Size	Number of patients or subjects included
Clinical Setting	e.g., ICU, ED, operating room, general ward, outpatient
Patient Characteristics	Age, sex, comorbidities, inclusion/exclusion criteria, disease group
Indication for VExUS	Clinical rationale or reason VExUS was applied
VExUS Details	VExUS version, grading system used, vessels assessed (IVC, hepatic, portal, intrarenal)
Operator & Training	Who performed ultrasound and their level of training/experience
Comparator Tool	Any tool or standard VExUS was compared to (e.g., CVP, biomarkers, other US methods)
Outcomes	e.g., AKI resolution, mortality, length of stay, and changes in diagnosis or treatment
Key Findings	Main quantitative or qualitative results
Author Conclusions	Summary of what the authors concluded about VExUS’s utility
Limitations	Limitations (reported by authors)
Notes	Space for reviewer comments, including questions, unusual findings, or incomplete data.

Each reviewer’s formal data extraction charts will be compared, and any disagreements will be discussed and resolved through consensus. Our third reviewer will adjudicate differences if no resolution is established.

Consistent with scoping review methodology, formal quality appraisal will not be conducted; however, study limitations and evidence gaps will be documented.

A finalised chart will be produced, for use in our data analysis and synthesis of results.

### Data analysis and presentation

Using the finalised chart, we will summarise findings related to the study characteristics and research questions. This will be summarised and presented using tables and narrative descriptions. Where appropriate, data will be grouped thematically by clinical setting, patient population, VExUS indication, and reported outcomes to identify patterns and knowledge gaps across contexts.

Given the anticipated heterogeneity of study designs, populations, and outcomes, a meta-analysis will not be appropriate. No formal statistical analyses will be conducted, as the purpose of this scoping review is to map and describe the existing body of literature on VExUS rather than to synthesise quantitative findings or estimate effect sizes.

### Study timeline

At the time of submission, this is a protocol for a scoping review study that has not yet commenced and has not generated results. No participant recruitment or primary data collection will take place, as these are not applicable to this study, where we will be extracting data based off existing research and literature. The planned timeline for the review is as follows: 3–4 weeks for record screening, 3–4 weeks for data extraction, 3 weeks for reviewing and summarizing findings, and 4–6 weeks for manuscript drafting. The study will begin after acceptance for peer review, pending any revisions that may arise from the review process. We anticipate that the final results will be ready within 3 months after study initiation.

## Discussion of limitations and impact

As a scoping review, this study will not include formal quality appraisal or risk of bias assessment. Additionally, despite efforts to ensure comprehensive coverage, some relevant studies may be missed due to indexing limitations or exclusion of non-English publications. The exclusion of non-English language studies may introduce language bias and limit global generalisability. Additionally, some relevant studies may use VExUS-related methods without explicitly naming the tool, which may lead to occasional omissions despite our focused keyword-based strategy.

Another limitation is the preliminary omission of forward and backward citation tracking. Although this is a deliberate methodological choice given the anticipated comprehensiveness and sensitivity of our multi-database and grey literature search, it may still result in missed studies, particularly those that are less well-indexed and cited. Selective citation chaining may be incorporated during the synthesis phase if key studies emerge.

Despite these limitations, we anticipate that this comprehensive scoping review will encourage broader consideration of VExUS use across diverse clinical contexts, supporting earlier detection and management of sequelae related to systemic venous congestion. In particular, expanding VExUS to non-acute settings may influence long-term management of chronic disease, reduce admissions, or optimise outpatient fluid management.

## Supporting information

S1 File PRISMA-ScR Checklist. The checklist used to ensure reporting standards for the scoping review.
(PDF)

S2 File PRISMA-P Checklist. The checklist used to ensure reporting standards for the protocol.
(PDF)
